# Methodologically grounded semantic analysis of large volume of chilean medical literature data applied to the analysis of medical research funding efficiency in Chile

**DOI:** 10.1186/s13326-020-00226-w

**Published:** 2020-09-29

**Authors:** Patricio Wolff, Sebastián Ríos, David Clavijo, Manuel Graña, Miguel Carrasco

**Affiliations:** 1 Business Intelligence Research Center, Universidad de Chile, Beauchef 851, Santiago, Santiago, 8370459 Chile; 2 Computational Intelligence Group, University of Basque Country, P. Manuel Lardizabal 1, San Sebastián, 20018 Spain; 3 Universidad Adolfo Ibañez, Santiago, Chile

**Keywords:** Data science, Machine learning, Latent Dirichlet allocation, Healthcare management, Strategy

## Abstract

**Background:**

Medical knowledge is accumulated in scientific research papers along time. In order to exploit this knowledge by automated systems, there is a growing interest in developing text mining methodologies to extract, structure, and analyze in the shortest time possible the knowledge encoded in the large volume of medical literature. In this paper, we use the Latent Dirichlet Allocation approach to analyze the correlation between funding efforts and actually published research results in order to provide the policy makers with a systematic and rigorous tool to assess the efficiency of funding programs in the medical area.

**Results:**

We have tested our methodology in the Revista Médica de Chile, years 2012-2015. 50 relevant semantic topics were identified within 643 medical scientific research papers. Relationships between the identified semantic topics were uncovered using visualization methods. We have also been able to analyze the funding patterns of scientific research underlying these publications. We found that only 29% of the publications declare funding sources, and we identified five topic clusters that concentrate 86% of the declared funds.

**Conclusions:**

Our methodology allows analyzing and interpreting the current state of medical research at a national level. The funding source analysis may be useful at the policy making level in order to assess the impact of actual funding policies, and to design new policies.

## Background

Due to the speed of innovation and change of research trends in the medical community, research topic taxonomies published by governmental agencies for funding calls often diverge from the reality of the research practice. Our working hypothesis is that semantic topic analysis provides an unbiased and accurate portrait of the actual research topics that are generating published results. In this paper we exploit the information from a national medical publication, described below, to identify the areas of active research, correlating them with the acknowledged funding sources, and non-funded personal effort backing these scientific results. This analysis provides the policymaker with a systematic, unbiased, and automated tool for the evaluation of the results of funding programs, allowing to assess the coherence of the national research funding policies with the actual research outcomes.

### Methodology background

The growth of number of PubMed references (from 363 in 2009 to 1820 in 2019 in a search with the terms “biomedical literature analysis”) demonstrates the increasing research efforts devoted to the automatic process of the biomedical literature, starting with the clustering of documents dealing with the same issues [1] based on the identification of semantic relevant terms, which can either be defined by some preexisting ontology or provided by a human expert. Document clustering is fundamental for online indexing of biomedical publications allowing easy search based on supervised machine learning approaches [2] in order to decrease human processing costs and increase availability of semantically indexed documents to the research community. Natural language processing techniques are useful for the extraction of information for further processing, such as ranking of terms in order to discover new concepts like phenotypic disease characterization [3]. Recent works use deep learning techniques to anchor a specific semantic ontology in the relevant literature [4]. A very promising application of medical literature is the discovery of new relations between concepts that may lead to breakthrough treatments [5].

The definition of the semantic domain is the first step in any attempt to automatic biomedical literature processing. The identification of topics for document semantic indexing can be done by humans that carry out the manual annotation of documents. Another approach is the so called topic modelling, i.e. the automated induction of semantic topics from the document data, under the assumption that these topics are defined in a latent space which can be uncovered by analytical means. Topic modeling alleviates the cost in human resources and time of the semantic domain definition, but the discovered topics are not guided by any human medical expert meaning, hence they require post-hoc human validation and interpretation. Latent Dirichlet Allocation (LDA) is the foremost topic modeling approach. It has been applied on different types of documents and their corresponding knowledge disciplines (regardless of the format in which the information is found as longs as it is text), such as work place and personal e-mails, abstracts in scientific documents and newspapers [6]. LDA has allowed pattern discovery in words and documents in the medical field, where it has been used to link diagnostic groups, medicines and publications [7–16]. After the topic modelling achieved by LDA and post-hoc analysis of the discovered topics, some meta-analysis can be carried out over the topic segmentation of the semantic domain. In our study, we perform a descriptive statistical analysis of the declared funding sources, which allows to assess the impact of funding agencies in the research actually reported in the literature.

### Case study background: medical scientific research in Chile

We showcase our approach on the analysis of medical scientific production in Chile, using as the main information source for this task the *Revista Médica de Chile* (RevMed). RevMed is a national and international reference in terms of dissemination of knowledge in the medical area. It was founded in 1872 as a result of the creation of the *Sociedad Médica* (Medical Society) in 1869. It’s the third oldest periodical publication of Chile, it’s the oldest medical journal in South America and second oldest in Spanish language in the world [17], and still continues to be relevant.

RevMed has covered in depth the technological and knowledge progress in each of the main medical research areas, such as clinical research, public health, ethics, medical education, and medical history. Consequently, it has a very important role in educational tasks, learning and scientific knowledge development in the country. For these reasons it is a faithful record of medical research in Chile.

## Materials and methods

### Corpus

Our corpus is composed of 643 research papers from RevMed published between the years 2012 and 2015. Prior to 2012, there is no access to online documents that could be used in our experiments. Besides, our processing capabilities allowed us to process only until year 2015. The categories of the papers included in the corpus were: Research Papers, Review Papers, Special Paper and Clinical Cases. Public Health articles and Letters to the Editor sections were excluded. The distribution of the number of papers *per* issue is shown in Table 1.

**Table 1:** Monthly and annual distribution of the Research Articles downloaded from RevMed (2012-2015)

	**jan**	**feb**	**mar**	**apr**	**may**	**jun**	**jul**	**aug**	**sep**	**oct**	**nov**	**dec**	**total**
**2012**	16	15	16	15	17	15	14	12	10	16	13	11	**170**
**2013**	14	15	16	12	13	13	12	13	14	14	14	15	**165**
**2014**	13	15	13	14	14	13	8	13	14	13	14	14	**158**
**2015**	13	12	11	13	14	13	14	14	12	12	12	10	**150**
**total**	**56**	**57**	**56**	**54**	**58**	**54**	**48**	**52**	**50**	**55**	**53**	**50**	**643**

### Methodological steps

**Fig. 1:**
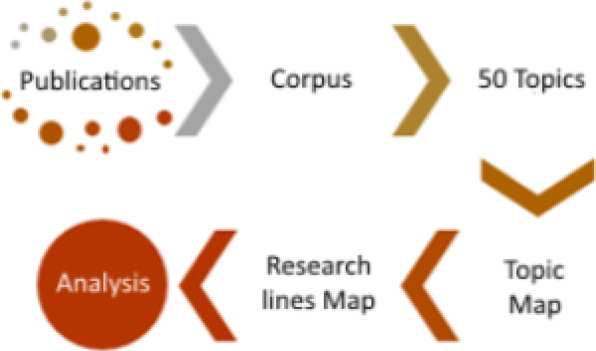
Methodological steps for the analysis of medical research funding from the literature

Figure 1 visualizes the methodological steps followed by our research funding analysis based on the semantic analysis of medical literature. The steps of the methodology are the following:

1. Publications: We carried out the textual data preprocessing, which consisted in cleaning and removing less relevant content in order to select meaningful words in the medical context that allow to provide interpretations to the topics identified by LDA.
  - Every character of the articles was converted to lowercase.
  - Non-alpha numeric characters were removed.
  - Double spaces between words were removed.
  - Numbers were removed. Even if some entity names may contain numbers, this has no impact on our methodology as far as we are looking for high level semantic topics.
  - Words or terms irrelevant for our analysis were removed, such as prepositions, conjunctions, articles, etc. Additionally, terms with very high frequency were removed (rev, med, Chile, etc).
  Preprocessing of documents was done on the programming language R (version 3.3.0), using the tm library (version 0.6-2) [18, 19], which provides a very convenient text mining framework.
2. Corpus: We extract the latent topics from the preprocessed corpus through LDA probabilistic modeling using the R package topicmodels (version 0.3-2)[20]. We use three different metrics, i.e. Griffiths2004 [14]; CaoJuan2009 [21]; and Arun2010 [22], to evaluate the quality of the topic modeling in order to determine the optimal number of topics for the ensuing semantic analysis. Each topic is represented by its 30 more meaningful words.
3. Topics: We give an interpretation and name to each of the LDA identified topics using the topic visualization tool LDAvis (version 0.3.2) [23]^1^ running in R, D3,^2^ and the qualitative analysis of a team of medical experts. The team was composed of three medical staff from the local hospital with extensive experience publishing in the journal, and research experience to carry out the topic semantic identification. Funding sources were not considered in this process. Additionally, we achieve an information size reduction going from the number of papers to the number of topics.
4. Topic Map: Using the visualization tool LDAvis, we create a 2D map of the LDA identified topics, where we were able to identify groups of topics by the judgment of experts, achieving a further information dimensionality reduction from the number of topics to the number of groups groups. Additionally, the axes of the topic map were interpreted accordingly to the topic grouping.
5. Research lines map: Publications or research papers where assigned to each of the research lines to observe its scientific production.
6. Funding Analysis: We use the funding acknowledgements in each paper to compute statistics of funding *per* research topic and research line.

### Latent Dirichlet allocation

Relevant definitions

- A document is a sequence of *N* words denoted by *w*=(*w*_1_,*w*_2_,…,*w*_*N*_), where *w*_*n*_ is the *n*−*th* word of the sequence
- A corpus is the collection of *M* documents denoted by *D*={*d*_1_,*d*_2_,…,*d*_*M*_}

Latent Dirichlet Allocation (LDA) is a generative probabilistic model of a corpus, where every document of the corpus is represented as a mixture of latent topics, and each topic is characterized by a probability distribution of words [24–26]. Specifically, in LDA this probability distribution is a Dirichlet distribution [27].

**Fig. 2:**
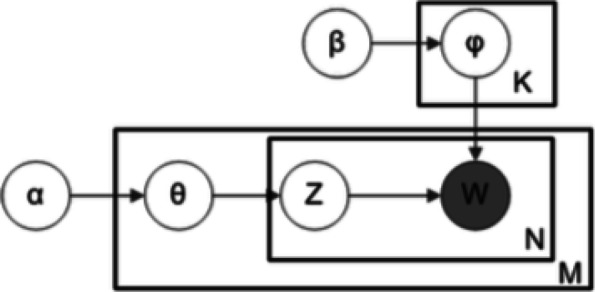
LDA’s model graphic representation

The model is represented with plate notation in Fig. 2. The parameters *α* and *β* are the priors of the Dirichlet distributions of topics per document and word per document, respectively. The inner and outer rectangular plates represent the word positions in a document and the documents, respectively. Each word position is associated with a topic choice *z*_*ij*_∈{0,1}. Each document *d*_*i*_ is described by a distribution of topics *θ*_*i*_. Additionally, each topic *k* is modelled by a distribution of words *φ*_*k*_, where we have a total of *K* topics. Equation (1) summarizes the model.

1 $$ p\left(\theta,z,w |\alpha,\beta\right)=p\left(\theta |\alpha\right) \prod\limits_{n=1}^{N}p\left(z_{n} |\theta\right)p\left(w_{n} | z_{n},\beta\right)  $$

The generative process underlying LDA for each document of the corpus is as follows:

1. Choose a N ∼ Poisson (*ξ*):
2. Choose a *θ*∼ Dir (*α*)
3. For each of the words *w*_*n*_ of N:
  - Choose a topic *z*_*n*_∼ Multinomial (*θ*)
  - Choose a word *w*_*n*_ according to *φ*(*w*_*n*_|*z*_*n*_,*β*), a conditional probability on the topic *z*_*n*_.

Where N follows a Poisson distribution with mean *ξ*, and *θ* and *φ* follow a Dirichlet distribution with parameters *α* and *β*, respectively.

### Topic model visualization systems

There are many systems and applications for the visualization of the results of topic modeling, (Termite [28], MALLET^3^ [29], ThemeRiver [30], and FacetAtlas [31] to name a few). Most of them try to link documents, topics and words for deeper post-hoc analysis of the obtained topic modelling results. The representations used by these systems are several: a list of words that belong to topics, limited bar graphs associated with the frequency of these words, clouds of relevant words describing a topic, pie charts representing the probability of each topic in a document, and many others.

For our work we have selected LDAvis [23], a visualization tools that allows quick and easy understanding of the modeling results. It carries out multidimensional scale analysis, achieving a distribution in a bidimensional space of the topics each represented by a circle. The size of a topic circle represents the relevance of the topic within the entire corpus, and each topic is associated to a list of relevant words describing it. The distance in the bidimensional projection space between the circle centers is a measure of the similarity of the topics: more similar topics have their circles placed at shorter distances. This tool allows to describe the meaning of each topic; to determine the prevalence of each topic in the corpus, and to infer the similarity link between each of the obtained topics.

## Results

### Topic modeling implementation

**Fig. 3:**
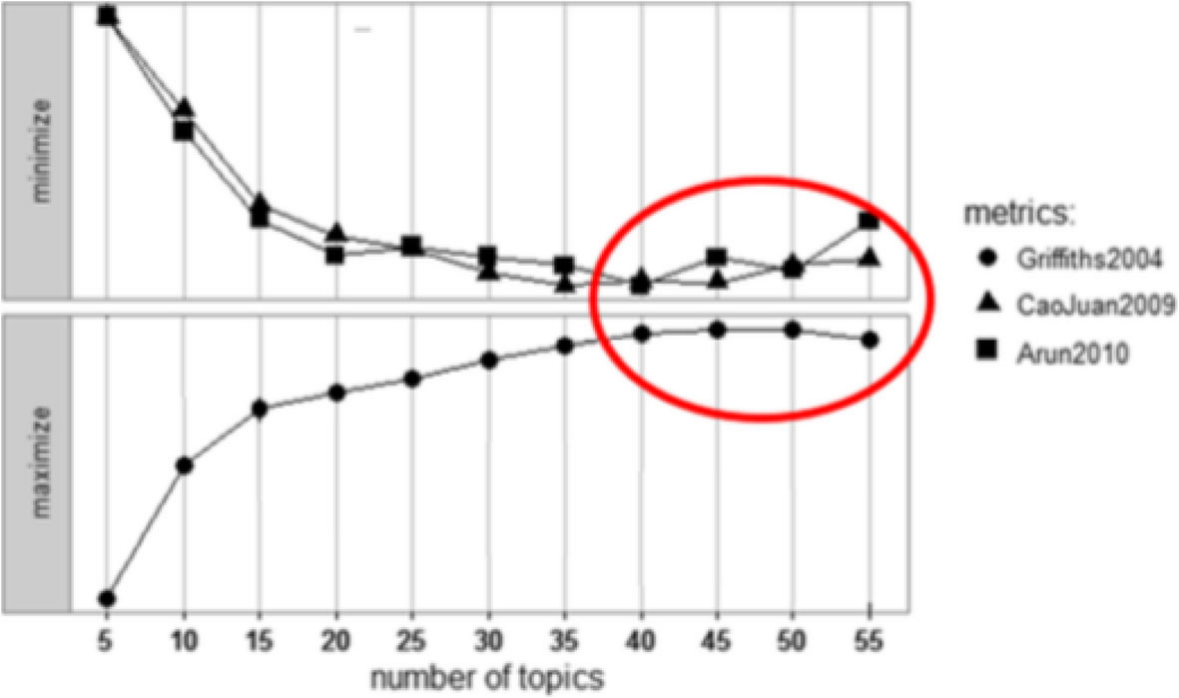
Plot of the metrics for the identification of the optimal number of topics for the analysis. The upper part corresponds to the metrics that are minimized, the lower part for the metric that is maximized

To estimate the optimal quantity of topics, we explored the results of topic modeling on the processed corpus carrying out a grid search over the number of topics from 5 up to 55. Figure 3 shows the results of this exploration. The optimal number of topics corresponds to the minimal values of Griffiths2004 and CaoJuan2009 metrics, and the maximal value of the Arun2010 metric. According to the plots in Fig. 3, it can be inferred that the optimal number of topics is K=50. After finding out the optimal number of topics, we apply two topic modeling approaches: Latent semantic analysis (LSA) and LDA. We apply Gibbs’s sampling [32] to estimate the parameters and inference. We used the gensim Python libraries,^4^ and the R implementations of LDA and LDAvis [23] for our purposes.

**Fig. 4:**
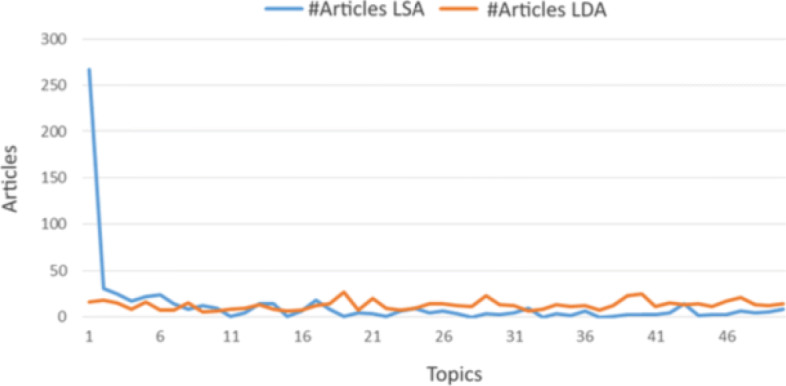
Distribution of the number of documents *per* topic for LDA and LSA algorithms

Comparing topic modeling results of the LSA and LDA approaches, LDA achieved better results according to the distribution of the number of documents *per* topic shown in Fig. 4. One of LSA topics accumulated 42% of the total research articles. Such concentration hinders the analysis and doesn’t allow to make meaningful interpretations. For this reason, we selected LDA results for deeper analysis and interpretation.

For LDA, the number Gibbs’s sampling scans over the whole corpus was set as 5000. The scalar value of Dirichlet distribution hyperparameter for the word distribution *per* topic, and for the topic distribution *per* document was set to 0.02.

**Fig. 5:**
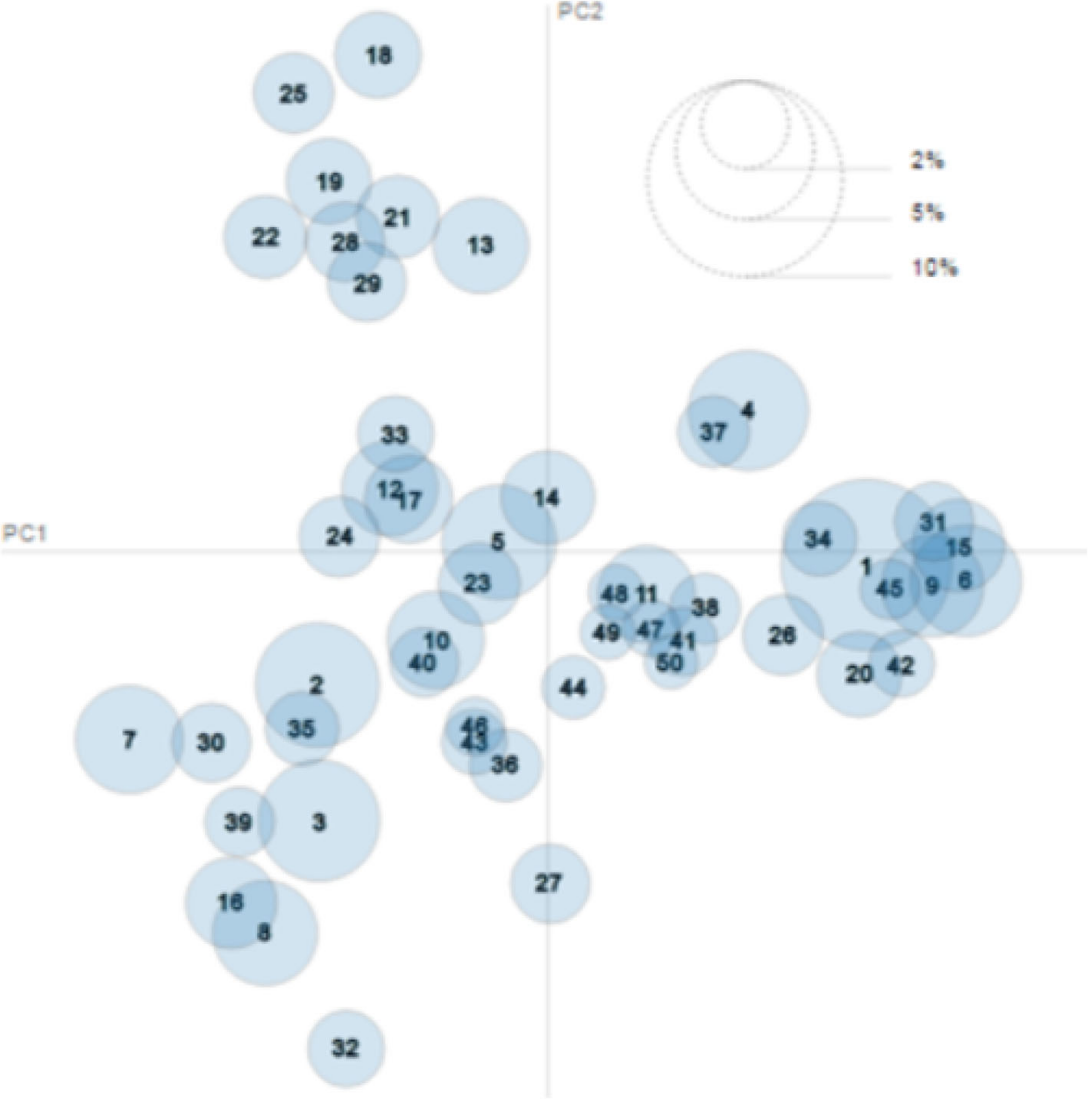
Visualization of the 50 most relevant topics found by LDA in the corpus. Circle diameter is proportional to topic relevance

LDA results visualization using LDAvis places topics discovered by LDA in a 2D space spanned by the principal components found by multidimensional scaling. In this visualization the Euclidean distance between the centers of the topic circle representation is a measure of the similarity between topics. This visualization also allows a quick inspection of the association between words and topics for a qualitative assessment of each topic meaning. The visualization of the topic spatial distribution can be observed on Fig. 5. Each topic is represented by a circle whose center is determined a multidimensional scaling process [33, 34] computed over the distances between topic word distributions. The prevalence of each topic is visualized via the proportional size of the circle diameters. The axes of the bidimensional map are constructed from the main components that come from the multidimensional scaling reduction of dimensionality process.

### Topic modeling term results

**Fig. 6:**
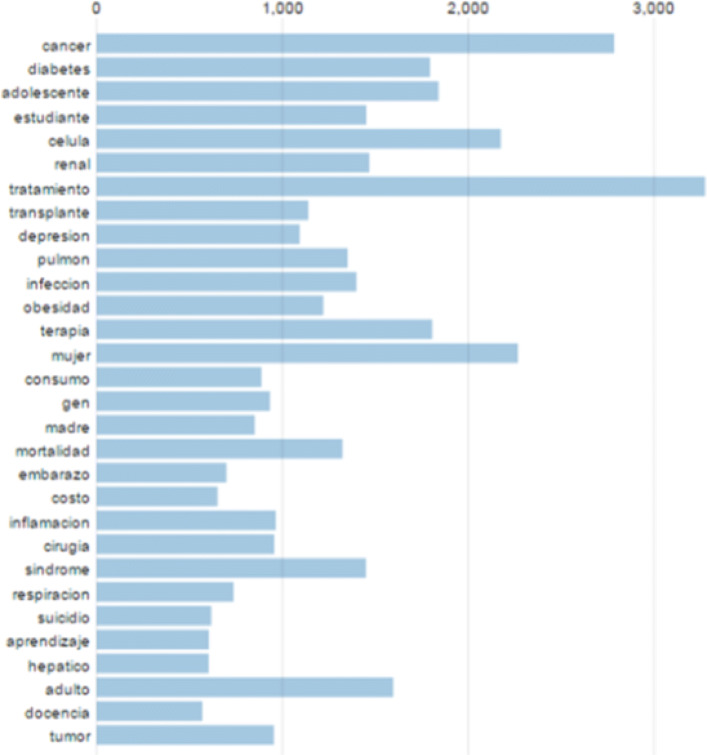
The 30 most frequent terms of the corpus

The corpus vocabulary is composed of 12,328 different terms, after removing repetitions, we got a total of 307883 unique terms. We discarded terms with less than 5 repetitions. The most frequent terms of the entire corpus can be observed in Fig. 6. It can be observed that terms such as treatment, cancer, woman and cell, are found more than 2,000 times in the corpus

### Expert post-hoc analysis of the results

**Fig. 7:**
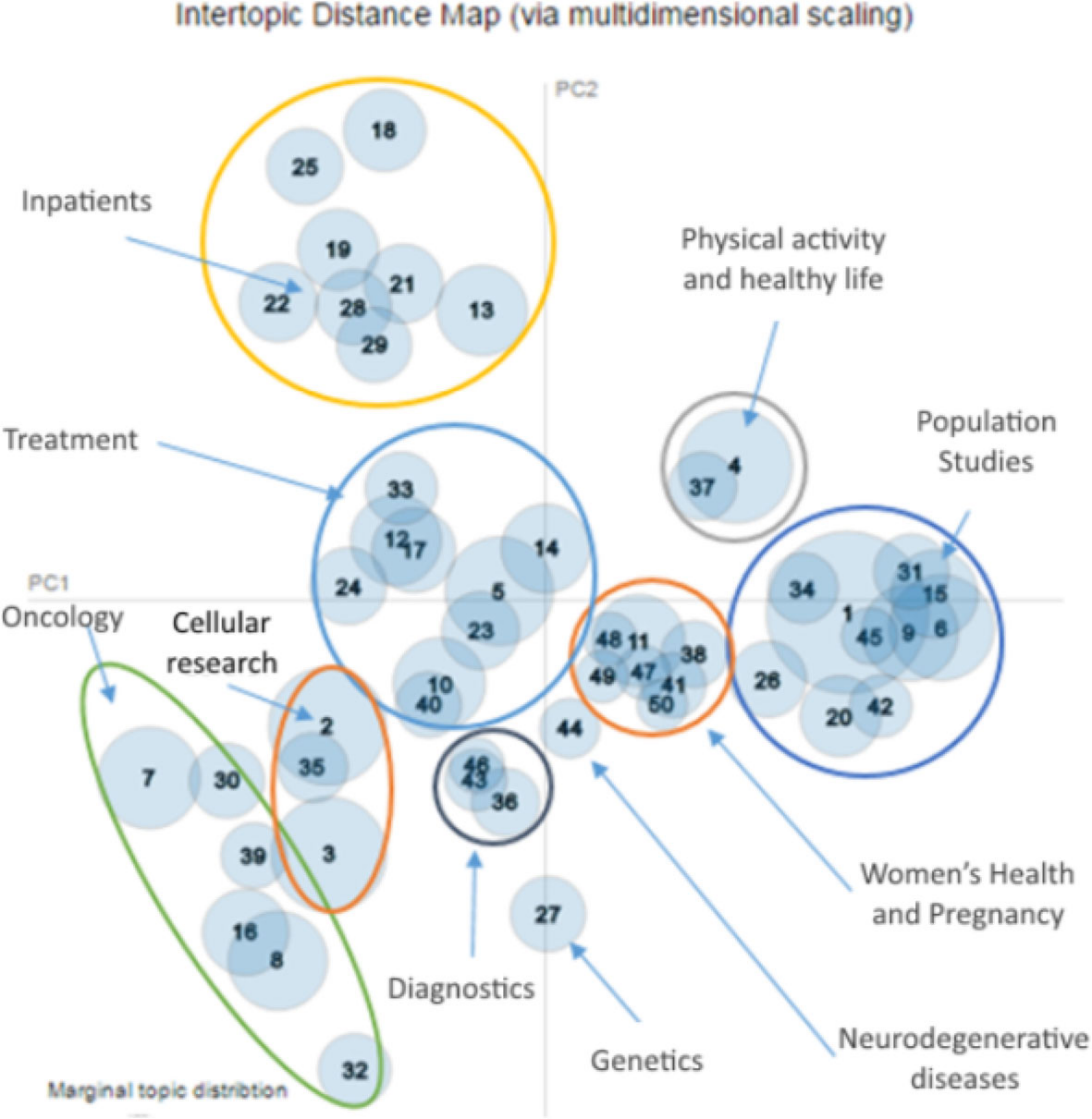
Visualization of topic grouping done by the team of experts based on semantic grounds

The judgment of experts was used to give a semantic interpretation clustering the 50 topics into groups with similar semantics. The result of this grouping was 11 clusters of topics, each one identified with its name as shown on Fig. 7. The group name identifies its general medical area, according to the meaning of the common terms found in the aggregated topics. It is possible to find interesting topics in the bidimensional space that are far away from the others, such as topic 32, which is located on the lower left side of the map (encompassing 1.5% of corpus terms) but that nevertheless semantically belong to a group. This topic is too specific when taking into account its representative terms, but they allow to aggregate it into the oncology group because it is related to cancer genetic studies. The oncology cluster is elongated in the representation space, due to the specificity of its belonging topics. Some clusters are single topics, like topic 27 containing genetics research. Topic 4 located in the upper right zone (encompassing 3.8% of corpus terms), almost defines a single topic cluster specifically devoted to physical activity and healthy life research.

The grouping of the topics allows us to give a semantic interpretation to the bidimensional space in which the topics are located. The horizontal axis corresponds to the size of the population under study in the reported medical research, ranging from research on individual subjects up to the entire population (going from the left to the right of the map). The vertical axis corresponds to the stage of the research according to the management of the disease, ranging from evaluating hypotheses about its cause and describing diagnostic instruments up to its prognosis or management (going from the bottom to the top of the map). The map origin is the location of studies associated with treatments and patient care.

### Topic modeling application to the analysis of research funding sources

The papers in the corpus describe research works that were developed with or without direct funding. Funding sources could be public, private or mixed. We found that only 29% of the researches published in RevMed in the period 2012-2015 declared direct funding sources, where the nature of the declared funding source is distributed as follows: 62% public, 32% private, and 6% mixed public and private. Some groups of topics concentrate the highest amount of funds: 87% of the funds were distributed into five groups: Treatment (22%), Oncology (20%), Inpatient research (18%), Population Studies (16%) and in Women’s Health and Pregnancy (11%). These groups cover a large fraction (over 85%) of the published papers in this period.

**Fig. 8:**
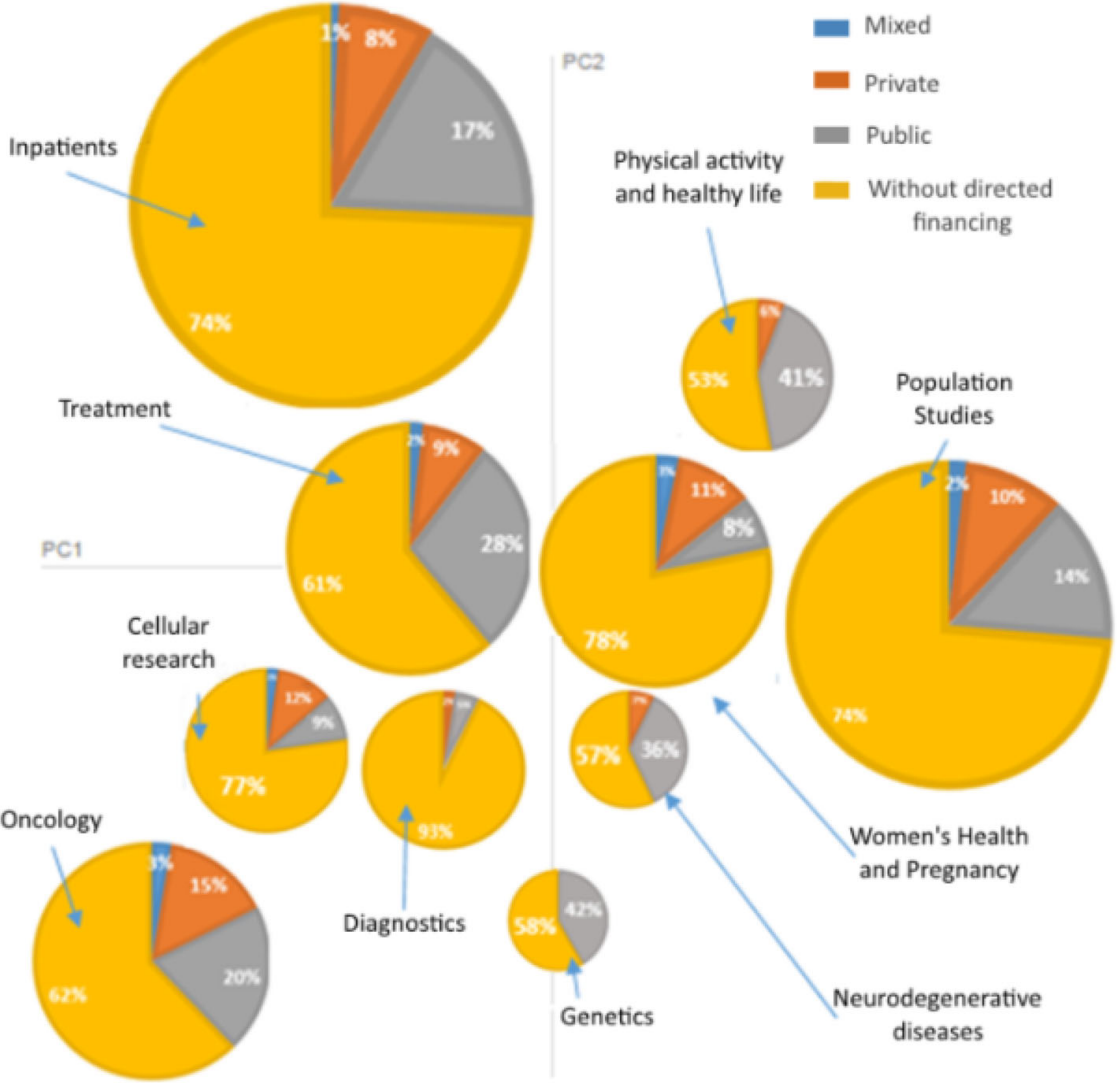
Nature of funding sources for each group of topics

We visualize the funding sources in the topic distribution bidimensional space, as shown in Fig. 8. Each pie represents the funding source distribution in each group of topics. The size of each circle represents the quantity of research works reported in each group of topics.

## Discussion

### Regarding the techniques used

The document analysis methodology presented in this paper is structured in a sequence of stages which achieve a progressive dimensionality reduction of the data arriving to a visual representation that allows diverse semantic-drive analysis by experts. We use well known techniques and software tools allowing the analysis of large volumes of data with off-the-shelf computing resources.

### Regarding the number of topics

We have carried out a systematic search for the optimal number of topics in the sense of three metrics that are well known in the literature. The search was carried out incrementally. In our computational experience, the effect of small increments of the number of topics is difficult to ascertain, hence we have carried out jumps of size 5 in the exploration. The determination of the optimal number is carried out detecting when the three measures start to degrade, which in our study happens after 50 topics. Though it may be argued that a more fine exploration would be advisable, we note that the ensuing semantic interpretation gives the meaning to the topics and determines their usefulness for the desired analysis. Each topic is described in such a specific manner by its representative words that they were easy to identify by experts. Out of the 50 topics, medical experts found difficulties in giving a semantic interpretation only for one of the topics (topic 48). Moreover, we are looking for big research categories, hence we want to avoid over-segmentation of the semantic space into small categories that would clog the analysis.

### Regarding processing time

The data processing time, from the topic modeling algorithm application up to the visualization of topics in the bidimensional space, was approximately 22 minutes using a laptop with an Intel Core i7 processor. Though this processing time is affordable for the considered corpus, scalating the methodology to extremely big corpuses like PubMed would require much bigger computing resources and some rewriting of the code to allow for parallel execution of several threads. One of the time critical tasks is the preprocessing of the corpus, which can be easily formulated as a trivially parallel task.

### Regarding the funding efficiency

A very salient conclusion is that most of the research in the medical area that achieves publication is done by researchers not involved in directly funded projects, overall only 29% of the papers report funding sources). This percentage is quite homogeneous, so it seems that funding is not a driver of published research. We report the relative percentages of the events ‘funded’ versus ‘non-funded’, but we do not have at this time information about the amounts of the acknowledged projects. This information would allow to compute research efficiency measures like the investment of money leading to each publication, and their differences among medical specialities. Another issue of interest is the relation between the prevalence and cost of the diseases and the cost of each publication. Unfortunately, at the time of writing we do not have this information, but it is an avenue of research worth pursuing.

Regarding funded research, we found that almost all groups of topics, except for groups with low scientific production (10 or less papers), receive similar funding measured in number of funded paper publications. On the other hand, there are strong differences in the publishing productivity among topics.

### Regarding the generalization of the methodological approach

The basic methodological approach can be applied to diverse fields of science, and even documented industrial activity. However, our intermediate results can not be translated directly. In several of the methodological steps, a team of experts is required, i.e. to do the semantic identification of the LDA topics, and to provide interpretations of the visualization map axes and groupings of topics. Therefore, these intermediate results are only meaningful in the framework of the current study.

## Conclusions

We have analyzed 643 scientific papers (2012-2015) of the chilean medical journal RevMed using topic modelling followed by topic visualization that reflects the topics in which medical research is being carried out in the country and their degree of funding. This analysis allowed to reduce gradually the quantity of information from the orginal 643 scientific documents, to 50 topics described by some 30 words each, down to the aggregation into 10 groups of topics located in the visualization space. Finally we are able to identify the meaning of the axes of the bidimensional space in which the topics where located as the size of the population under study (horizonatal axis) and the stage of the research carried out (vertical axis). A team of experts was able to interpret each topic representative words in order to assign them a specific semantic. The same was done for each group of topics and the dimensions of the bidimensional visualization space.

This study shows the application of text mining techniques in medical knowlege areas whose results can be utilized for socio-economical analysis of the research activity in the medical area, specifically we demonstrated that it provides tools to evaluate the impact of funding policies on the published research. Future work will be addressed to gather additional information in order to assess the specific funding resources backing each publication and its correspondence with the real needs of the population in terms of disease prevalence and estimated cost.

## Abbreviations

D3: data driven document visualization
LDA: Latent Dirichlet Allocation
LDAvis: LDA visualization tool
LSA: Latent Semantic Analysis
Python: Programming language
R: The R Project for Statistical Computing
RevMed: Revista Medica de Chile

## Footnotes

1 https://pyldavis.readthedocs.io

2 https://d3js.org

3 https://wp.nyu.edu/exceltextanalysis/visualize-mallet-topics/

4 https://radimrehurek.com/gensim/
